# Temporal Trends and Clinical Implications of Cardiac Troponin Testing in Emergency Departments: A Multicenter Retrospective Study

**DOI:** 10.3390/jcm15062426

**Published:** 2026-03-22

**Authors:** Jong-Ho Kim, Youngho Seo, Seung Yong Shin, Eung Ju Kim, Kap Su Han, Hyung Joon Joo

**Affiliations:** 1 Korea University Research Institute for Medical Bigdata Science, Korea University, Seoul 02842, Republic of Korea; 2 Department of Cardiology, Korea University Ansan Hospital, Ansan 15355, Republic of Korea; 3 Department of Cardiology, Korea University Guro Hospital, Seoul 08308, Republic of Korea; 4 Department of Emergency Medicine, Korea University Anam Hospital, Seoul 02841, Republic of Korea; 5 Department of Cardiology, Korea University Anam Hospital, Seoul 02841, Republic of Korea

**Keywords:** acute coronary syndrome, troponin, risk assessment, emergency service

## Abstract

**Background:** Cardiac troponin testing is central to evaluating suspected acute coronary syndromes, yet its expanding use may increase resource utilization in low-risk emergency department populations. **Methods:** We conducted a multicenter retrospective cohort study across three tertiary hospitals in South Korea (2017–2023) using harmonized electronic health record data integrated with the Observational Medical Outcomes Partnership Common Data Model (OMOP-CDM) and the National Emergency Department Information System (NEDIS). Visits were stratified into low, intermediate, and high risk by age and chest pain presentation, and cardiac troponin T was categorized as normal (<0.014 ng/mL), borderline (0.014–0.052 ng/mL), or elevated (>0.052 ng/mL). Outcomes included emergency department length of stay, hospital admission, 30-day revisit, 30-day coronary revascularization, and 30-day mortality. **Results:** Among 727,772 visits, troponin testing increased from 29.8% in 2017 to 45.5% in 2023. High-risk patients were consistently tested, whereas testing rose substantially in intermediate- and low-risk groups. In high-risk patients, normal troponin values were associated with lower 30-day revascularization and mortality, without prolonging length of stay or increasing admissions. In contrast, in lower-risk groups, testing was associated with longer stays and higher admissions without clear short-term clinical benefit. **Conclusions:** These findings support more targeted troponin testing protocols to optimize emergency department resource use while preserving diagnostic performance in higher-risk presentations.

## 1. Introduction

Cardiac troponins are structural proteins of the thin filament regulatory complex in cardiomyocytes, released into the circulation following myocardial cell injury or necrosis. Their highly tissue-specific expression and prolonged elevation after myocardial damage have established them as the preferred biomarkers for diagnosing acute myocardial infarction [1,2]. Over the past two decades, their adoption has expanded exponentially, facilitating early risk stratification and guiding clinical decision-making [3]. However, concerns have emerged regarding potential overuse and the misapplication of troponin testing to populations at low risk for ACS, leading to unnecessary resource utilization and increased healthcare costs [4,5]. Recent studies indicate that a significant subset of troponin tests in the emergency department (ED) may not alter patient management or improve outcomes, underscoring the need for more judicious testing protocols [6,7].

While previous studies have examined the diagnostic yield of troponin testing in ACS [8], few have investigated temporal trends in testing practices and their association with clinical endpoints such as revascularization rates and mortality. In many EDs, patients present with incomplete or nonspecific symptoms, prompting clinicians to order troponin tests “just in case,” particularly when managing borderline or atypical presentations. This practice may subject low- or intermediate-risk individuals to unnecessary testing, prolonged ED stays, and unwarranted admissions, while offering uncertain benefits in terms of short-term mortality and invasive interventions. Furthermore, the clinical implications of normal troponin results in different risk groups have not been well characterized in large-scale studies.

We hypothesized that the clinical utility of troponin testing varies significantly by risk category and that current testing practices may not align optimally with patient risk profiles. Using standardized data from three tertiary hospitals in South Korea, we aimed to evaluate temporal trends in cardiac troponin testing, analyze testing patterns across risk categories, and identify potential overuse among lower-risk populations, and determine whether normal troponin results in high-risk patients can expedite ED processes and reduce hospital admissions. Ultimately, our findings seek to inform more targeted troponin utilization strategies that preserve diagnostic accuracy for ACS while curbing unnecessary resource consumption and patient burden.

## 2. Materials and Methods

### 2.1. Study Design, Setting, and Data Source

This multicenter, retrospective cohort study was conducted using the Observational Medical Outcomes Partnership Common Data Model (OMOP-CDM), the National Emergency Department Information System (NEDIS) and hospital information system databases from three tertiary hospitals (Anam hospital, Guro hospital and Ansan hospital). The OMOP-CDM, developed by the Observational Health Data Sciences and Informatics (OHDSI) collaboration, provided a standardized framework for structuring electronic health records (EHRs) [9]. Clinical data, including diagnoses coded according to the International Classification of Diseases, 10th Revision (ICD-10), medications, and laboratory results, were harmonized into unique concept identifiers within the OMOP-CDM. Detailed emergency medical information was extracted using the NEDIS database of each hospital. The NEDIS is a nationwide government-managed registry that collects standardized clinical data from 402 EDs across South Korea [10]. Additional data not covered by the OMOP-CDM or the NEDIS, such as electrocardiography, were directly extracted from the research database of the EHRs of each hospital. All extracted data were stored in Microsoft SQL servers and accessed through structured SQL queries.

### 2.2. Selection of Patients

Data of the patients who visited the ED of each hospital between January 2017 and December 2023 were extracted. Patients who were transferred to the other hospital or died in the ED or stayed in the ED less than 15 min or more than 72 h were excluded. Finally, data of 163,975 patients (238,258 cases) in Anam hospital, 187,722 patients (270,913 cases) in Guro hospital, 151,314 patients (218,601 cases) in Ansan hospital were extracted and analyzed further.

### 2.3. Risk Stratification

Given the limitations of EHR data for detailed chest pain characteristics, we developed a simplified risk stratification algorithm based on two fundamental parameters: chest pain presentation and age. While established tools like the Emergency Department Assessment of Chest Pain Score (EDACS) demonstrate superior performance in prospective settings, their retrospective application using EHR data remains challenging due to incomplete documentation of pain characteristics and impractical for patients without chest pain [11].

Patients were classified into three risk groups:

- High-risk: Patients presenting with chest pain-specific chief complaint codes and age >45 years.
- Low-risk: Patients without chest pain codes and age ≤45 years.
- Intermediate-risk: All remaining patients, including those with chest pain but age ≤45 years, or without chest pain but age >45 years.

### 2.4. Analysis

Continuous variables were presented as mean ± standard deviation and categorical variables as numbers with percentages. Temporal trends were assessed using Kendall’s tau correlation coefficient. The Kruskal–Wallis test was used to compare continuous variables across groups, with post hoc pairwise comparisons performed using Bonferroni correction. For categorical variables, chi-square tests were conducted with pairwise nominal independence tests using Bonferroni adjustment for multiple comparisons.

For risk stratification analysis, patients were classified into three risk categories (low, intermediate, and high) based on predefined criteria. Cardiac troponin T (cTnT) values were categorized as normal (<0.014 ng/mL), borderline (0.014–0.052 ng/mL), or elevated (>0.052 ng/mL). Clinical outcomes included ED length of stay, hospital admission, 30-day ED revisit, 30-day revascularization, and 30-day mortality. Hospital admission was defined as inpatient admission occurring at the conclusion of the index emergency department visit, and did not include subsequent readmissions during the 30-day follow-up period. Levene’s test was performed to assess homogeneity of variance across groups. All statistical tests were two-sided, and *p*-values <0.05 were considered statistically significant. All analyses were carried out using R Statistical Software version 4.1.2 (R Foundation for Statistical Computing, Vienna, Austria).

## 3. Results

**Figure 1:**
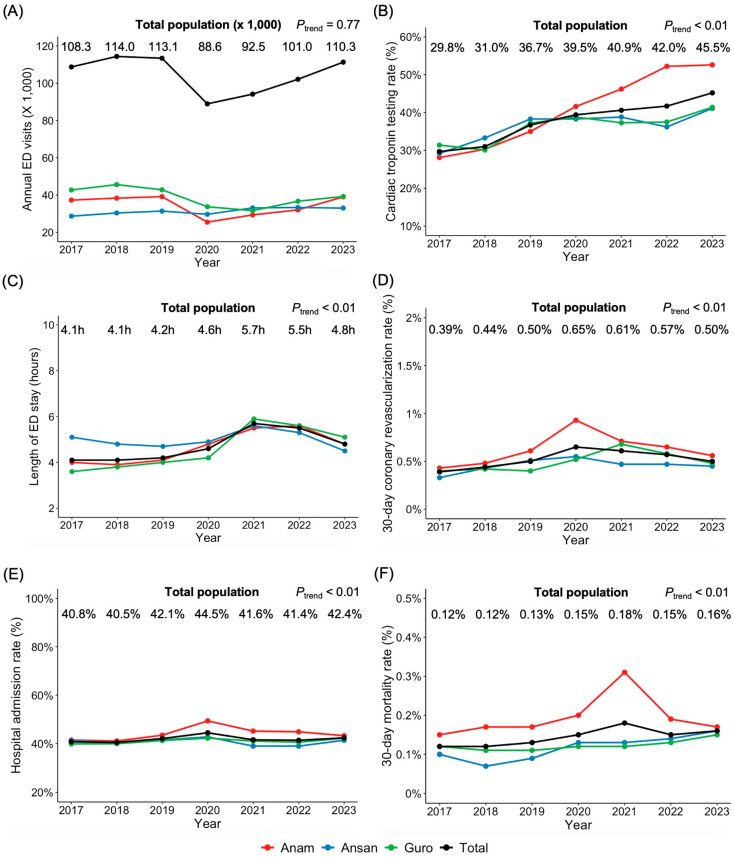
Temporal trends in annual emergency department visits, cardiac troponin testing rates, and key clinical outcomes from 2017 to 2023 in three hospitals. (**A**) Annual number of emergency department (ED) visits (in thousands). (**B**) Annual proportion of ED patients who underwent cardiac troponin testing. (**C**) Length of ED stay. (**D**) 30-day coronary revascularization rates. (**E**) Annual hospital admission rates. (**F**) 30-day mortality rates. Data points represent annual values from 2017 through 2023 for Anam hospital (red), Ansan hospital (blue), Guro hospital (green), and total population (black). Trend analysis was performed using Kendall’s tau test.

Between 2017 and 2023, a total of 727,772 ED visits were recorded across three tertiary hospitals (Figure 1). The annual ED visit volume showed minimal fluctuation from 2017 to 2019 (108,272 to 113,055 visits), followed by a transient decline in 2020 (88,617 visits) attributable to the COVID-19 pandemic, with subsequent recovery to pre-pandemic levels by 2023 (110,348 visits). The trend analysis revealed no significant overall trend (*p* for trend = 0.77). In contrast, the proportion of patients undergoing cardiac troponin testing rose from 29.8% in 2017 to 45.5% in 2023 (*p* for trend < 0.01). The median ED length of stay increased overall from 4.1 ± 4.2 h in 2017 to 4.8 ± 5.5 h in 2023 (*p* for trend < 0.01). Hospital admission rates grew slightly from 40.8% in 2017 to 42.4% in 2023 (*p* for trend < 0.01). Thirty-day coronary revascularization rate also rose from 0.39% to 0.50% (*p* for trend < 0.01), while 30-day mortality rate increased from 0.12% to 0.16% (*p* for trend < 0.01). These findings indicate that expanded cardiac troponin testing was accompanied by longer emergency department stays and modest increases in admission rates, revascularization procedures, and short-term mortality at the population level. Whether this expansion translated into proportional clinical benefit may differ according to patient risk profile.

**Figure 2:**
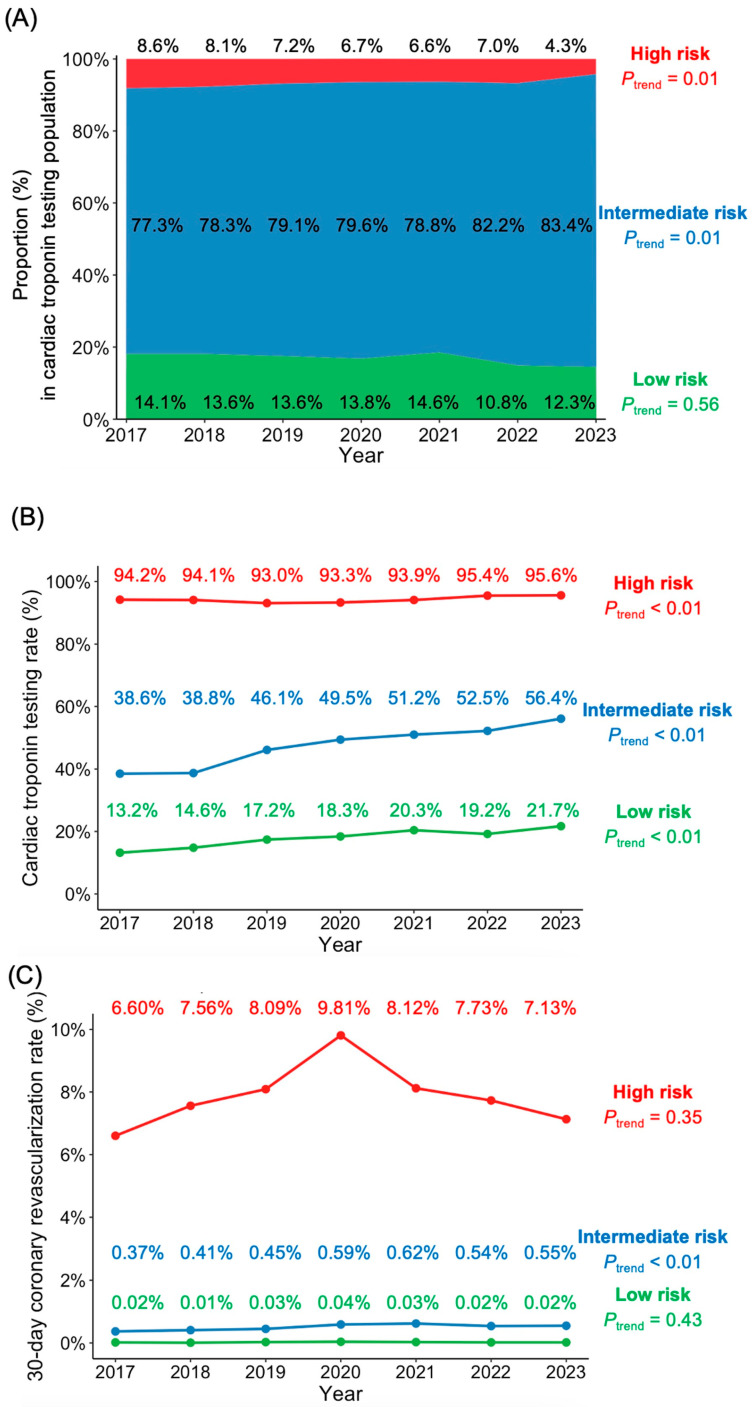
Annual distribution of risk category, cardiac troponin testing rates, and 30-day revascularization from 2017 to 2023. (**A**) Annual proportion of patients classified as high risk (red), intermediate risk (blue), or low risk (green) among those who underwent cardiac troponin testing. Percentages for each group are shown. (**B**) Cardiac troponin testing rates by risk category. Each line depicts the percentage of patients tested within that category for each year. (**C**) 30-day coronary revascularization rates stratified by the same risk categories. Data points represent annual values from 2017 through 2023. All *p*-values were calculated using the Kendall’s tau test for trend.

From 2017 to 2023, 242,077 patients (33.3%) underwent cardiac troponin testing. Using a pragmatic risk stratification algorithm based on age and chief complaint, we classified patients into three risk categories. Among troponin-tested patients, the proportion classified as intermediate risk increased from 77.3% in 2017 to 83.4% in 2023 (*p* for trend = 0.01), while the high-risk group decreased from 8.6% to 4.3% (*p* for trend = 0.01) (Figure 2A). The low-risk category showed a non-significant decline from 14.1% to 12.3% (*p* for trend = 0.14).

Troponin testing patterns varied markedly by risk category (Figure 2B). High-risk patients maintained consistently high testing rates, increasing slightly from 94.2% to 95.6% (*p* for trend < 0.01). The intermediate-risk group demonstrated a substantial increase in troponin utilization from 38.6% to 56.4% (*p* for trend < 0.01), while the low-risk group, despite lower overall testing frequency, showed a significant relative increase from 13.2% to 21.7% (*p* for trend < 0.01).

The 30-day coronary revascularization rates differed significantly across risk groups (Figure 2C). Low-risk patients maintained minimal revascularization rates throughout the study period (*p* for trend = 0.43). In the intermediate-risk group, revascularization rates increased modestly from 0.37% to 0.55% (*p* for trend < 0.01). High-risk patients exhibited relatively higher revascularization frequencies, ranging from 6.60% to 9.81%, without significant linear trend (*p* for trend = 0.35).

Despite the rise in troponin use, the overall proportion of patients undergoing revascularization remained modest, suggesting that many individuals—particularly in low- and intermediate-risk categories—may receive troponin tests without a corresponding likelihood of invasive intervention. This marked disparity between troponin testing frequency and subsequent revascularization rates highlights the need for more refined risk stratification algorithms and testing protocols to better target troponin evaluation toward patients with the highest likelihood of benefiting from intervention.

Patients stratified by cTnT levels demonstrated significant differences in clinical outcomes. Among 727,772 ED visits, the majority of patients had no troponin test performed (66.7%), followed by normal cTnT (<0.014 ng/mL, 19.9%), borderline (0.014–0.052 ng/mL, 9.2%), and elevated (>0.052 ng/mL, 4.2%). ED stay length progressively increased across cTnT categories, with the longest duration observed in patients with elevated cTnT (>0.052 ng/mL; 9.9 ± 9.1 h), followed by borderline (0.014–0.052 ng/mL; 8.3 ± 7.4 h), normal (<0.014 ng/mL; 6.0 ± 5.3 h), and untested groups (3.5 ± 3.8 h; *p* < 0.01, Table 1). Hospital admission rates showed a similar stepwise increase, from 30.8% in the untested group to 50.1%, 81.6%, and 92.4% in the normal, borderline, and elevated cTnT groups, respectively (*p* < 0.01). The 30-day coronary revascularization rate increased substantially with higher cTnT levels, from 0.1% in the untested group to 0.5%, 1.3%, and 5.2% in the normal, borderline, and elevated groups, respectively (*p* < 0.01). The 30-day mortality rates also showed a marked increase with rising cTnT levels, from 0.1% in the untested group to 0.8% in the elevated group (*p* < 0.01). These findings suggest a clear association between higher cTnT levels, longer ED stays, increased likelihood of hospital admission, the need for coronary interventions, and higher short-term mortality.

Among patients stratified by risk category and troponin testing status, normal troponin results demonstrated varying clinical implications. In the high-risk group (chest pain with age > 45 years), patients with normal troponin values exhibited similar mean ED stay length and hospital admission rates compared to untested high-risk patients. Notably, patients with normal troponin values had lower 30-day revascularization and mortality than untested high-risk patients. These findings suggest potential benefits of confirming normal troponin values in high-risk populations.

In contrast, normal troponin results in low- and intermediate-risk patients did not demonstrate similar advantages. These groups experienced paradoxically longer ED stays and higher admission rates compared to untested patients, despite normal biomarker values. Although tested patients with normal results showed marginally higher 30-day revascularization and mortality rates compared to untested patients, the absolute numbers were extremely small, indicating limited clinical relevance. These observations suggest that routine troponin testing in low- and intermediate-risk populations may increase healthcare resource utilization without appreciable short-term clinical benefits, while normal troponin results in high-risk patients may reduce invasive coronary revascularization and short-term mortality.

## 4. Discussion

In this large-scale multicenter study of 727,772 ED visits from 2017 to 2023, we observed a significant increase in cardiac troponin testing rates (29.8% to 45.5%) despite stable ED visit volumes, with notable variations across risk categories. While high-risk patients maintained consistently high testing rates (94.2% to 95.6%), we found substantial increases in testing among intermediate-risk (38.6% to 56.4%) and low-risk groups (13.2% to 21.7%). Importantly, normal troponin results showed contrasting clinical implications across risk categories: in high-risk patients, normal troponin levels were associated with reduced 30-day revascularization and mortality rates compared to non-testing, without prolonging ED stays or increasing hospital admission rates, whereas in low- and intermediate-risk patients, testing was paradoxically associated with longer ED stays and higher admission rates without substantial clinical benefits. The 30-day revascularization rates remained disproportionately low relative to the volume of troponin testing, particularly in low-risk patients, suggesting potential overutilization of troponin testing in this population. These findings highlight the need for more targeted testing strategies based on patient risk profiles to optimize resource utilization while maintaining diagnostic efficacy.

This aligns with recent evidence showing that patients with undetectable high-sensitivity troponin have up to 20% lower mortality risk compared to the general population [12]. The benefit of confirming normal troponin in high-risk patients likely reflects standardized chest pain protocols and clear clinical pathways that enable confident rule-out of acute coronary syndrome.

In contrast, the limited clinical advantages observed in low- and intermediate-risk patients with normal troponin results suggest potential overtesting in these populations. Our results extend previous evidence regarding cardiac biomarker overuse in low-risk populations. Makam et al. have shown that indiscriminate troponin testing in low-probability populations may result in substantial downstream resource utilization without proportionate clinical benefits [13]. Wassie et al. demonstrated that patients discharged after a single negative troponin test had similar 30-day outcomes to those undergoing serial testing, suggesting limited value of routine testing in low-risk groups [14]. The 2021 AHA/ACC guidelines specifically recommend against routine troponin testing in low-risk patients with previous normal evaluations [15]. The marked increase in troponin testing we observed among intermediate-risk patients (38.6% to 56.4%) despite minimal changes in revascularization rates (0.37% to 0.55%) particularly exemplifies this concern, supporting the findings that expanded biomarker testing may not necessarily improve patient outcomes.

Our findings also suggest that more targeted testing strategies, such as incorporating the HEART score or similar risk stratification tools, could optimize resource utilization without compromising patient outcomes [16,17]. Furthermore, adopting a single undetectable high-sensitivity troponin measurement as sufficient for rule-out in low-risk presentations may reduce the need for serial testing and prolonged observation without compromising diagnostic safety. This is particularly relevant given evidence that indiscriminate troponin testing in low-risk populations may paradoxically increase healthcare utilization through additional downstream testing and prolonged observation periods.

The discrepant outcomes between risk groups may be explained by several mechanisms. In high-risk patients presenting with chest pain, troponin testing likely prompted more comprehensive clinical evaluations and expedited decision-making through standardized chest pain protocols, an approach supported by evidence that troponin-integrated rule-out strategies reduce unnecessary admissions in symptomatic populations [18]. This structured approach, supported by evidence that troponin-based protocols can safely expedite disposition in patients presenting with suspected ACS [19,20], may explain the absence of prolonged ED stays in our high-risk group despite high testing rates. Conversely, in low- and intermediate-risk patients, troponin testing often occurs without systematic protocols, potentially leading to diagnostic uncertainty and prolonged observation despite normal results.

Provider behavior and institutional factors may also contribute to these differences. Recent studies suggest that emergency physicians may order troponin tests “just in case” in lower-risk patients, particularly when presenting symptoms are ambiguous or when there is pressure to expedite patient flow [21,22]. This defensive testing strategy, combined with the lack of clear guidelines for interpreting normal troponin results in low-risk populations, may paradoxically lead to additional diagnostic testing and longer ED stays [23,24]. Furthermore, the presence of comorbidities, which are more common in intermediate-risk patients, can complicate the interpretation of troponin results and influence clinical decision-making, potentially contributing to more conservative management strategies despite normal biomarker values [25,26].

Our study has several important limitations. First, the retrospective design using EHR data may have limited our ability to capture detailed clinical information such as specific chest pain characteristics that could influence risk stratification. Second, our simplified risk stratification, while practical for large-scale analysis, may not fully account for other important clinical factors that physicians consider when ordering troponin tests. Third, the study was conducted at three tertiary hospitals in South Korea, potentially limiting the generalizability of our findings to other healthcare settings or populations. Fourth, we could not account for all potential confounding factors that might influence testing decisions and outcomes, such as physician experience, departmental protocols, or local practice patterns. Fifth, the observational nature of our study precludes establishing causal relationships between troponin testing patterns and clinical outcomes, and we did not examine parallel trends in other routine diagnostic modalities such as electrocardiography. Despite these limitations, our large sample size and standardized data collection across multiple centers provide valuable insights into real-world troponin testing practices and their clinical implications.

## 5. Conclusions

Cardiac troponin testing in the ED has expanded substantially over time, yet its clinical benefit varies markedly by patient risk profile. These findings support a shift toward more targeted, risk-stratified testing protocols that preserve diagnostic value in high-risk presentations while reducing unnecessary resource utilization in lower-risk populations.

## Figures and Tables

**Table 1:** Clinical Outcomes According to cTnT Categories in Emergency Department Patients.

Total population	Not Tested N = 485,695	Normal cTnT N = 154,042	Borderline cTnT N = 66,793	Elevated cTnT N = 30,242	*p*-Value
Length of ED stay, hours	3.5 ± 3.8	6.0 ± 5.3	8.3 ± 7.4	9.9 ± 9.1	<0.01
Hospital admission rate (%)	149,395 (30.8)	72,635 (50.1)	54,483 (81.6)	27,957 (92.4)	<0.01
30-day coronary revascularization rate (%)	562 (0.1)	730 (0.5)	888 (1.3)	1584 (5.2)	<0.01
30-day mortality rate (%)	355 (0.1)	132 (0.1)	308 (0.5)	249 (0.8)	<0.01
**Low risk**	**Not tested** **N** = 236,510	**Normal cTnT** **N** = 27,913	**Borderline cTnT** **N** = 2646	**Elevated cTnT** **N** = 1261	***p*** **-value**
Length of ED stay, hours	3.1 ± 3.2	5.8 ± 5.4	8.0 ± 7.4	9.5 ± 9.1	<0.01
Hospital admission rate (%)	49,726 (21.0)	9759 (35.1)	1878 (71.0)	1108 (87.9)	<0.01
30-day coronary revascularization rate (%)	10 ( < 0.1)	14 (0.1)	9 (0.3)	27 (2.1)	<0.01
30-day mortality rate (%)	21 ( < 0.1)	14 (0.1)	4 (0.1)	7 (0.6)	<0.01
**Intermediate risk**	**Not tested** **N** = 246,383	**Normal cTnT** **N** = 107,292	**Borderline cTnT** **N** = 60,433	**Elevated cTnT** **N** = 26,201	***p*** **-value**
Length of ED stay, hours	3.8 ± 4.2	6.1 ± 5.4	8.4 ± 7.5	10.1 ± 9.1	<0.01
Hospital admission rate (%)	98,308 (39.9)	58,237 (54.3)	49,777 (82.4)	24,247 (92.5)	<0.01
30-day coronary revascularization rate (%)	427 (0.2)	395 (0.4)	488 (0.8)	893 (3.4)	<0.01
30-day mortality rate (%)	328 (0.1)	116 (0.1)	293 (0.5)	214 (0.8)	<0.01
**High risk**	**Not tested** **N** = 2802	**Normal cTnT** **N** = 9837	**Borderline cTnT** **N** = 3714	**Elevated cTnT** **N** = 2780	***p*** **-value**
Length of ED stay, hours	5.5 ± 7.1	5.3 ± 3.9	6.8 ± 5.8	8.1 ± 8.7	<0.01
Hospital admission rate (%)	1361 (48.6)	4603 (46.8)	2828 (76.1)	2602 (93.6)	<0.01
30-day coronary revascularization rate (%)	125 (4.5)	321 (3.3)	391 (10.5)	664 (23.9)	<0.01
30-day mortality rate (%)	6 (0.2)	2 (<0.1)	11 (0.3)	28 (1.0)	<0.01

Data are presented as mean ± standard deviation or number (%). Normal cTnT is defined as <0.014 ng/mL, borderline as 0.014–0.052 ng/mL, and elevated as >0.052 ng/mL. *p*-values were calculated using one-way ANOVA for continuous variables and chi-square test for categorical variables. All statistical tests showed significant differences between groups (*p* < 0.01).

## Data Availability

The data and codes utilized or analyzed in this study are accessible from the corresponding author upon request.

## Abbreviations

ACS	Acute coronary syndromes
ED	Emergency department
OMOP-CDM	Observational Medical Outcomes Partnership Common Data Model
NEDIS	National Emergency Department Information System
ICD-10	International Classification of Diseases, 10th Revision
cTnT	Cardiac troponin T
EDACS	Emergency Department Assessment of Chest Pain Score
